# “Aging Means to Me… That I Feel Lonely More Often”? An Experimental Study on the Effects of Age Simulation Regarding Views on Aging

**DOI:** 10.3389/fpsyg.2022.806233

**Published:** 2022-02-28

**Authors:** Laura I. Schmidt, Anna Schlomann, Thomas Gerhardy, Hans-Werner Wahl

**Affiliations:** ^1^Institute of Psychology, Heidelberg University, Heidelberg, Germany; ^2^Institute for Educational Sciences, Heidelberg University of Education, Heidelberg, Germany; ^3^Network Aging Research, Heidelberg University, Heidelberg, Germany

**Keywords:** age simulation, awareness of age-related change, aging-related cognitions, age stereotypes, risk perception, technology acceptance, geriatric assessments, views on aging

## Abstract

Over the last decades, educational programs involving age simulation suits (ASS) emerged with the ambition to further the understanding of age-related loss experiences, enhance empathy and reduce negative attitudes toward older adults in healthcare settings and in younger age groups at large. However, the impact of such “instant aging” interventions on individuals’ personal views on aging have not been studied yet. The aim of the current study is to address possible effects of ASS interventions on multiple outcomes related to views on aging, i.e., aging-related cognitions (i.e., expectations regarding social losses), awareness of age-related change (AARC) and age stereotypes. Moreover, we explore effects on broader constructs with relevance to aging, i.e., perceived obsolescence, risk perceptions, as well as desired support through technology. In a within-subjects design, N = 40 participants (*M* = 61.4 years, *SD* = 6.16) went through a series of established geriatric assessments (i.e., Timed up and Go) with and without an ASS. Views on aging constructs were assessed in standardized questionnaires before and after the ASS intervention. Changes in aging-related cognitions were observed, with more negative expectations regarding social integration and continuous development after wearing the ASS. AARC and age stereotypes did not change from pre- to post-assessment, but participants reported an increased susceptibility to age-associated impairments and stronger feelings of obsolescence. Those participants who exhibited higher difficulties in geriatric assessments while wearing the suit reported higher openness to be supported by intelligent assistive devices or robots afterwards. We conclude that ASS interventions should only be combined with education on losses and gains during the aging process to prevent negative effects on individual views on aging. On the other hand, potentials regarding technology acceptance and formation of intentions to engage in prevention and health behaviors among middle-aged to young-old adults are discussed.

## Introduction

First prototypes of age simulation suits (ASS) were constructed in the 1990s, with pioneers in the automotive industry aiming to raise engineers’ awareness of age-related impairments when designing new cars. Nowadays, a variety of suits is commercially available or can be rented for educational or training purposes. Typically, ASS (i.e., AGNES, GERT, Koken LM60, Sakamoto M176) combine features that simulate sensory decline, such as goggles, gloves, and hearing protectors, with devices that simulate musculoskeletal changes, such as weights and restrictors.

In the recent two decades, a relatively large body of rather descriptive studies has emerged containing mostly positive experiences with the application of ASS. Target populations predominantly included student groups in health-related professions; outcomes represented the areas of better understanding of what aging means, empathy with older adults, and attitudes toward older adults. Two recent review articles (Eost-Telling et al., 2020; Bowden et al., 2021) have targeted these psychological outcomes of age simulation in a broader approach by including variations of interventions that either used a “full” ASS, only single components (i.e., only goggles), or aging games including role-play instructions. In this study, we use the ASS as an *age simulation intervention* able to provide first-hand experiences of the motor-sensory deficits associated with advancing age for our participants (see also the definition of age simulation intervention in Bowden et al., 2021). In their review, Bowden et al. (2021) identified mainly positive effects of aging simulations on knowledge, empathy levels, and attitudes towards older adults among younger healthcare professionals. Similarly, Eost-Telling et al. (2020) focused on health and social care students and concluded that effects of such aging simulation interventions on attitudes towards older people were predominantly positive. Although both reviews concluded that such ASS interventions might be useful, for example to promote person-centered care, they also found large heterogeneity in the methodological quality among included studies. For example, the three randomized controlled trials that have been published until now were all among student populations and yielded mixed results (Mohamed et al., 2017; Cheng et al., 2020; Lee and Teh, 2020). Mohamed et al. (2017) reported that students who participated in an ASS intervention showed increased knowledge regarding typical changes associated with aging, less negative attitudes, but no significantly increased positive attitudes (measure: Kogan’s Attittudes Toward Older People Scale; KAOP; Kogan, 1961) in comparison to a control group that received a lecture on age-related changes. Cheng et al. (2020) used a control group wearing placebo clothes (i.e., a white wig) and found increases in positive attitudes (KAOP) in both groups, but no group differences favoring the ASS group. Similarly, Lee and Teh (2020) did not find group differences between students in the ASS intervention group and control group (polypharmacy workshop) regarding self-rated empathy levels (measure: Jefferson Scale of Empathy-Health Profession Students; Fields et al., 2011).

In addition to conflicting results in studies with established trial designs, existing studies largely focus on attitudes and empathy, whereas the effects of ASS on views on one’s own aging process such as awareness of age-related change (AARC; Diehl and Wahl, 2010), aging-related cognitions, or general age stereotypes in different life domains have to the best of our knowledge not been studied yet. The consideration of multiple indicators of views on aging as outcomes of wearing an ASS is important, because it has been argued that grasping the subjective experience of getting older rather comprehensively must consider different areas in parallel and thus needs the application of multidimensional assessment instruments (Diehl et al., 2014).

Moreover, research has not addressed, whether wearing an ASS also affects general expectations about aging, i.e., health-related risk perception or the desired support through technology as the result of age-related functional impairment (i.e., robots, intelligent assistive devices).

Striving for a broader focus, we intend to investigate potential changes in perceptions of *obsolescence*, defined as a gradual loss of social integration and perceived lack of competence to deal with the demands of modern society (Brandtstädter and Wentura, 1994; Kaspar, 2004), *risk perceptions* regarding age-related impairments and *desired support through robots or assistive technology*. With this broader approach aside explicit views on aging, we aim to explore potential future applications of ASS interventions. For example, perceived vulnerability or risk perception is an established predictor for intentions regarding health behaviors, i.e., to engage in physical activity (Schwarzer, 2001). Higher feelings of obsolescence have been linked to lower technology use, lower technology acceptance and worse performance with everyday technology (Kaspar, 2004; Schmidt and Wahl, 2019). On the other hand, the personal experience as “senior self” with the ASS and heightened awareness regarding possible challenges in physical tasks might also be a means to boost technology acceptance regarding assistive devices. Finally, and in contrast to previous studies that largely focused on young samples, we include middle-aged to “young-old” participants (Baltes and Smith, 2003) in order to address the aforementioned constructs that are related to successfully preparing for the aging process. Finally, studying views on aging through the lens of ASS might be especially important among middle-aged and young-old adults due to three primary reasons, following Lachman (2015): (1) Middle-aged individuals are in a phase of life that comes with first personal experiences of aging; (2) midlife is crucial for preparing the retirement transition and the subsequent post-work period frequently associated with the start of “old age”; and (3) midlife and young-old age seems to be a life phase with increased sensitivity for stereotypes about older adults and aging (Miche et al., 2014).

In the area of psychological aging research, the study of views on aging has developed both conceptually and empirically as a very promising field with relations to important developmental outcomes (Dutt et al., 2018). Views on aging can be understood as an umbrella term for (1) age stereotypes that relate to older adults as a social group without self-reference (Kornadt and Rothermund, 2011) and (2) personal or individual perceptions, experiences, and subjective beliefs or interpretations related to one’s own aging process (Steverink et al., 2001). Following Levy’s (2009) stereotype embodiment theory, both concepts are connected and affect life span development through different pathways. On the one hand, stereotypes influence behavior towards older adults (i.e., ageism), thereby creating a developmental context for older people (Kornadt and Rothermund, 2011). On the other hand, those general views on aging become part of a person’s self-concept and identity over time (self-stereotyping, internalization), which influences their attitudes toward their own aging (Levy, 2009). In the last two decades, research has shown a robust linkage between more negative views on aging on the one side and lowered subjective well-being, health, cognitive abilities, and longevity on the other side (Westerhof et al., 2014; Alonso Debreczeni and Bailey, 2021; Kaspar et al., 2021). With regard to the measurement of views on aging, it has been argued that unidimensional approaches and scales such as *attitudes towards own aging* (ATOA; Lawton, 1975) or single-item questions of *subjective age* neglect the potential that aging experiences might differ across different life domains. In contrast, a more recent multidimensional approach to measure individual views on aging is the concept of *awareness of age-related change* (Diehl and Wahl, 2010; Diehl et al., 2021) with ratings of positive and negative perceptions (AARC Gains and AARC Losses) in the five behavioral domains (1) health and physical functioning, (2) cognitive functioning, (3) interpersonal relations, (4) social-cognitive and social–emotional functioning, and (5) lifestyle and engagement. Similarly, approaches such as the *multidimensional ageing cognitions scales* (AgeCog; Steverink et al., 2001) ask for reflections of personal views on ageing regarding continuous growth, physical decline, and social losses.

Concluding, the aim of the present study was to examine whether possible effects of ASS interventions consistently generalize to a broad set of indicators representing a range of facets of individual and general views on aging. In contrast to recent findings among young participants indicating *positive* effects on attitudes or empathy towards older adults after an ASS intervention, we expected that views on one’s own aging process might change in a *negative* direction. Whereas earlier research has asked questions about older adults as an out-group (a social group with which a young individual does not identify, i.e., attitudes toward ‘them’), our middle-aged target group might be particularly sensitive for aging-experiences and interpret the ASS intervention as a ‘senior moment’ or ‘future me’. In detail, we assumed larger negative effects for those views on aging measures that depict situational aspects, namely aging-related cognitions. For those measures that are formed over longer time periods (awareness of age-related change) or are not self-referential (age stereotypes) we did not expect to find pre-to-post differences. In an explorative approach, we additionally addressed possible changes aside views on aging with respect to risk perception, obsolescence and technology acceptance, in order to explore potential future applications of ASS interventions.

## Materials and Methods

The study was pre-registered on OSF^1^ and followed APA ethical standards as well as the 1964 Helsinki declaration and its later amendments. The ethics commission of the Faculty of Behavioral and Cultural Studies at Heidelberg University, Germany, obtained ethical approval. In order to approach the statistical power for the planned pre-to-post comparisons in our within-subjects or repeated measures design with two measurement occasions, we approximated the effect size that can be detected with a power of 90% in our design using G^*^Power (Faul et al., 2009) for *t*-tests (dependent means). These analyses suggested that in order to detect medium-sized effects (Cohen’s *d* = 0.50), adequate power would be obtained with a minimum sample size of *N* = 36. All analyses (descriptive statistics, paired *t*-tests, (partial) correlations) were performed using SPSS version 25.

### Participants and Recruitment

Forty adults aged 51–72 years (*M*_age_ = 61.40, *SD* = 6.16; 9 men) participated in the age simulation study. All participants were recruited from a pool of inhabitants of the Rhine-Neckar region in Germany, that had already taken part in an unrelated online survey and had agreed to be contacted again for further studies. Of those, fifty-eight potential participants were contacted with information on study content and procedure. Forty-four individuals agreed to participate and were screened in a structured telephone interview. Due to our exclusion criteria (i.e., severe chronic diseases, mobility impairments or pain), four had to be excluded from further participation. Two individuals used a hearing aid, but all were able to understand the telephone-based screening questions uttered in natural voice with normal volume. Sociodemographic information and health-related information was collected and at the end of the screening, an appointment was arranged for the age simulation intervention at a motion capturing lab of Heidelberg university. The *N* = 40 participants who moved on from the telephone screening to the simulations rated their (corrected) vision as at least satisfactory (10%), good (68%) or very good (22%), had a BMI ranging from 19.49 to 35.43 (*M* = 24.76, *SD* = 3.94), did not report balance issues, and had not participated in a similar experiment before. All participants provided informed consent at the beginning and received 20 Euros at the end of the experiment.

### Age Simulation Intervention

In a within-subjects design, all participants underwent a series of established functional test (i.e., assessing balance, strength, gait parameters) that formed a comprehensive geriatric assessment with and without the age simulation suit GERT (www.produktundprojekt.de). The order of the conditions (with or without ASS first) was randomized: 21 participants were first assessed with the ASS followed by the same geriatric assessments without the ASS, 19 participants went through the geriatric assessments in reverse order (first without ASS, then with ASS). Participants were given approximately 5 min to get accustomed to the suit before the assessments started. On average, participants wore the ASS for 45 min. The GERT combines goggles, hearing protection, gloves, wrist and ankle weights, elbow and knee restrictors, a cervical collar, a weight vest, as well as overshoes meant to simulate an unstable gait. We conducted the Timed up and Go test (Podsiadlo and Richardson, 1991), the Short Physical Performance Battery (Guralnik et al., 1994), 30-s chair-stand test (Jones et al., 1999), Short Community Balance and Mobility Scale (Gordt et al., 2020) and a grip strength measure (JAMAR dynamometer; Mathiowetz et al., 1985).

### Pre- and Post-questionnaires

The following constructs were assessed in standardized pre- and post-questionnaires (paper and pencil) before and after the comprehensive geriatric assessment described above. More specifically, all participants (regardless of the order of assessments with and without ASS) first filled out the pre-questionnaires, were than randomly allocated to one of the two conditions (1. with ASS - without ASS or 2. without ASS – with ASS), and filled out the post-questionnaires after completion of all geriatric assessments. All internal consistencies of the applied questionnaires could be classified as at least acceptable with the exception of AARC Gains at pretest (Chronbach’s α = 0.56).

*Subjective Age* was assessed as a manipulation check using a single-item question at pre- and post-test (“How old do you feel?”) and while wearing the ASS (“How old do you feel with the suit?”) in order to provide data on the subjective aging effect induced by the suit.

*Aging-related cognitions* were assessed with the multidimensional AgeCog scales (Steverink et al., 2001). Following the intro “Ageing means to me that…” participants indicated the extent to which diverse statements reflected their own views on ageing regarding (1) physical decline (e.g., “…I am less energetic and fit,” Cronbach’s α_pre_ = 0.63, α_post_ = 0.79), (2) social losses (e.g., “…I feel lonely more often,” Cronbach’s α_pre_ = 0.62, α_post_ = 0.76), and (3) continuous growth/ongoing development (e.g., “…I can still learn new things,” Cronbach’s *α*_pre_ = 0.61, *α*_post_ = 0.68). Each scale consists of four respective items rated on a 4-point scale ranging from 1 (“strongly agree”) to 4 (“strongly disagree”). For each scale, scores were recoded and averaged with higher values indicating either more negative (Physical Decline and Social Losses) or more positive (Continuous Growth/Ongoing Development) views on one’s own aging process.

*Awareness of Age-Related Change* was assessed with the 10-item AARC short-form questionnaire (Kaspar et al., 2018) with the two dimensions Gains and Losses. Each item starts with the prompt “With my increasing age, I realize that…” followed by either a negative (e.g., “…my mental capacity is declining.”), or a positive experience (e.g., “…I appreciate relationships and people much more.”) in the five behavioral domains (1) health and physical functioning, (2) cognitive functioning, (3) interpersonal relations, (4) social-cognitive and social–emotional functioning, and (5) lifestyle and engagement. Items are rated on a 5-point Likert scale (1 = “not at all” to 5 = “very much”). Mean scores were computed with higher scores indicating more AARC gains (Cronbach’s *α*_pre_ = 0.62, *α*_post_ = 0.56) and AARC losses (Cronbach’s *α*_pre_ = 0.74, *α*_post_ = 0.71).

*Age stereotypes* were measured with two subscales of the multidimensional, domain-specific age stereotype scale (Kornadt and Rothermund, 2011). We assessed the domain of leisure activities and social/civic commitment (4 items), and the domain of physical and mental fitness, health and appearance (3 items). All items are assessed on 8-point rating scales that contrast two opposing statements, e.g., (1) “Old persons show commitment for others” vs. (8) “Old persons do not show commitment for others” or (1) “Old persons are rarely sick” vs. (8) “Old persons are sick a lot.” A mean score for both subscales was computed, ranging from 1 to 8, with higher scores indicating more favorable age stereotypes (Cronbach’s α leisure_pre_ = 0.83, leisure_post_ = 0.84, physical/health_pre_ = 0.74, physical/health_post_ = 0.66).

*Risk perception* was assessed using a measure of relative vulnerability according to guidelines proposed by Schwarzer (2001). In three items, participants were asked to rate their perceived risk regarding chronic pain, mobility limitations, and serious illnesses (Cronbach’s *α*_pre_ = 0.79, Cronbach’s *α*_post_ = 0.73) following the prompt “Compared to an average person of my sex and age my chances of getting X are...” with answers on a 5-point scale from 1 (“much below average”) to 5 (“much above average”).

*Perceived Obsolescence* was measured using the respective subscale from Brandstädter and Wentura’s questionnaire on experiencing time and future (1994). It consists of five items (e.g., “For me, life has become more and more complicated, more difficult to comprehend”; Chronbach’s *α*_pre_ = 0.72 and *α*_post_ = 0.73) with ratings on a 5-point scale from 1 (“not at all”) to 5 (“very true”).

*Desired support through technology,* a measure of technology acceptance, was assessed using three items originally designed for the German Aging Survey (“If needed, I would like to be supported by an assistive device or robot… in doing the housework… in taking medication… in body care.”; Cronbach’s *α*_pre_ = 0.85, Cronbach’s *α*_post_ = 0.74). Responses are given on a 4-point scale from 1 (“not at all”) to 4 (“very true”).

## Results

### Associations Between Study Variables at Baseline

Table 1 shows descriptive statistics and correlations among the study variables assessed at baseline. A higher age was related to more positive aging related cognitions (continuous growth: *r* = 0.33, *p* < 0.05; physical decline: *r* = −0.39, *p* < 0.05; social loss: *r* = −0.28, *p* = 0.08), and marginally to a higher risk perception (*r* = 0.28, *p* = 0.08). General age stereotypes and personal aging-related cognitions showed several significant associations, i.e., more positive general views regarding older adults in the leisure/social commitment domain and in the physical/health domain were related to higher agreement to participants’ own continuous growth (*r* = 0.42 and *r* = 0.47, *ps* < 0.01). With regard to health-related variables, those participants with a higher BMI and lower subjective health reported stronger agreement to the physical decline subscale (*r* = 0.33 and *r* = −0.35, *ps* < 0.05), as well as higher age-related losses (*r* = 0.36, *p* < 0.05 and *r* = −0.56, *p* < 0.001).

**Table 1:** Descriptive statistics and intercorrelations of the study variables.

S. no.	Variables	*M*	*SD*	2	3	4	5	6	7	8	9	10	11	12	13	14	15
1	Gender^a^	–	–	−0.46^**^	−0.27^+^	−0.43^**^	0.03	−0.03	0.07	−0.12	0.00	0.24	−0.14	−0.02	0.24	−0.04	0.23
2	Age	61.40	6.16		0.60^***^	0.06	0.15	0.09	0.21	0.33^*^	−0.39^*^	−0.28^+^	0.08	−0.15	−0.13	0.28^+^	−0.21
3	Subjective Age	52.05	9.85			0.04	0.08	0.06	0.41^**^	0.12	−0.21	−0.27^+^	0.12	0.04	−0.15	0.17	0.05
4	Body Mass Index^b^	24.76	3.94				−0.39^*^	0.02	0.03.	0.11	0.33^*^	0.07	0.29^+^	0.36^*^	0.21	0.13	−0.23
5	Subjective Health^c^	3.38	0.74					0.07	0.12	0.06	−0.35^*^	−0.05	−0.08	−0.56^***^	−0.51^***^	0.00	0.27^+^
6	Age stereotypes LC^d^	5.61	1.25						0.66^***^	0.42^**^	−0.24	−0.17	0.28^+^	−0.20	−0.09	−0.24	0.00
7	Age stereotypes PH^d^	4.79	1.26							0.47^**^	−0.44^**^	−0.25	0.30^+^	−0.23	−0.08	−0.14	0.15
8	AgeCog Continous Growth^e^	3.23	0.46								−0.26^+^	−0.47^**^	0.28^+^	−0.34^*^	0.00	−0.15	0.25
9	AgeCog Physical Decline^e^	2.78	0.60									0.42^**^	−0.13	0.48^**^	0.39^*^	0.00	−0.11
10	AgeCog Social Loss^e^	1.45	0.39										−0.25	0.27^+^	0.11	0.27^+^	−0.29^+^
11	AARC Gains^f^	3.66	0.63												0.09	0.15	−0.17
12	AARC Losses^f^	2.01	0.61												0.44^**^	0.25	−0.24
13	Risk Perception^g^	2.51	0.65													0.22	−0.06
14	Perceived Obsolescence^h^	1.87	0.54														−0.27^+^
15	Support through Technology^i^	2.03	0.93														–

**N* = 40, LC: leisure activities and social/civic commitment; PH: physical and mental fitness, health and appearance*.

a *0 = male, 1 = female, ^b^BMI; kg/cm^2^*.

c *1–5; higher scores indicate higher subjective health*.

d *1–8; higher scores indicate more positive views on aging*.

e *aging-related cognitions, 1–4, higher scores indicate stronger agreement*.

f *Awareness of age-related change, 1–5, higher scores indicate higher agreement*.

g *1–5, higher scores indicate higher perceived vulnerability in comparison to the same sex and age group*.

h *1–5; higher scores indicate higher obsolescence*.

i *1–4, higher scores indicate higher agreement*.

+ *p* *< 0.10;*

* *p* *< 0.05;*

** *p* *< 0.01;*

*** *p < 0.001*.

Desired support through technology, as a measure of technology acceptance, only showed marginally significant correlations with views on aging measures (Table 1). Participants who reported higher agreement to the social loss subscale and perceived higher obsolescence showed lower acceptance regarding support via assistive technologies. In more specific analyses we controlled for age (as older adults tended to exhibit lower acceptance) and differentiated between the areas of medication, household and body care. We found that higher perceptions of continuous growth were associated with higher overall agreement to be supported through assistive technology (*r* = 0.35, *p* < 0.05) as well as in the domain of medication (*r* = 0.35, *p* < 0.05) and household (*r* = 0.42, *p* < 0.01). In contrast, more negative aging-related cognitions regarding physical decline and higher age-related losses were associated with lower desired support in the body care domain (*r* = −0.36, *r* = −0.35, *ps* < 0.05) and negative cognitions regarding social loss were related to lower desired support in medication (*r* = −0.31, *p* = 0.05), household (*r* = −0.49, *p* < 0.01), and overall acceptance (*r* = −0.37, *p* < 0.05).

### Effects of the Age Simulation Intervention on Multiple Views on Aging and Related Domains

To quantify the effect due to the ASS, we calculated difference scores with respect to subjective age (age with ASS – subjective age) and chronological age (age with ASS – chronological age). Descriptive statistics on age measures with and without ASS are depicted in Table 2. On average, participants felt 9 years younger that their chronological age at baseline (subjective age: *M* = 52.5, *SD* = 9.6), although the range was large (28 years younger to 10 years older). While wearing the ASS, mean felt age was 81.1 years (*SD* = 9.7), again with a large interindividual variation. Compared to their chronological age, participants subjectively “aged” on average 19.7 years (*SD* = 9.3), and compared to their subjective age at baseline the aging effect covered 29.3 years (*SD* = 12.9). At post-test, participants subjective age dropped back and did not differ from baseline level (*M* = 53.3, *SD* = 9.4; *t* = −1.37, *p* = 0.18).

**Table 2:** Age measures and effect of the age simulation suit.

S. no.	Variables	Min	Max	*M*	*SD*
1	Chronological age	50	75	61.4	6.3
2	Subjective age	30	69	52.5	9.6
3	Subjective age – chron age	−28	10	−9.0	7.9
4	Subjective age with suit	50	100	81.1	9.7
5	Subjective age with suit – chron. age^a^	−9	37	19.7	9.3
6	Subjective age with suit – subj. Age	2	60	29.3	12.9

*N = 40*.

a *One woman was feeling younger with the suit in comparison to her chronological age (but not her subjective age), as excluding her from all analyses did not change results, we decided to report results with the full sample*.

**Figure 1:**
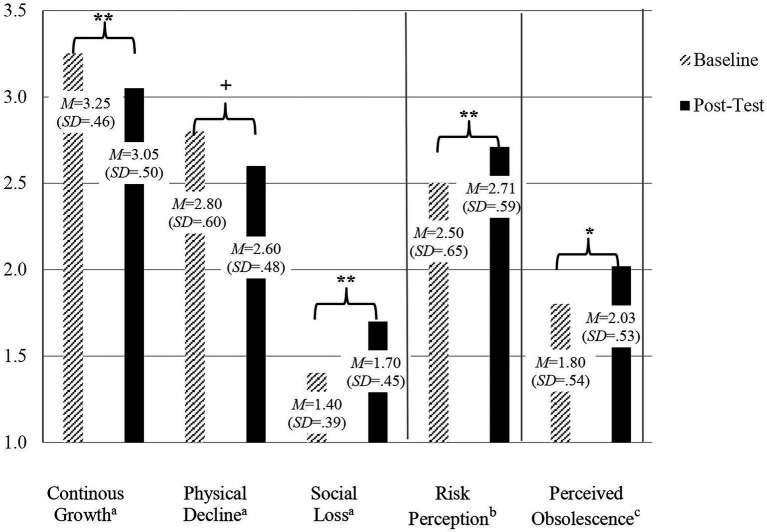
Change in ageing-related cognitions, risk perception and obsolescence from baseline to post-assessment following the age simulation intervention. *N* = 40; ^a^aging-related cognitions (AgeCog scale), 1–4, higher scores indicate stronger agreement; ^b^1–5, higher scores indicate higher perceived vulnerability in comparison to the same sex and age group; ^c^1–5; higher scores indicate higher obsolescence; ^+^*p* < 0.10; ^*^p < 0.05; ^**^p < 0.01.

To explore possible changes in views on aging, we conducted paired comparisons of baseline and post-assessments scores. Awareness of age-related change (gains and losses) and general age stereotypes (leisure/social commitment and physical/health domain) did not change from pre- to post-assessment (*ps* > 0.05). In contrast, and in accordance with our hypothesis regarding the more situational AgeCog scales, more negative cognitions regarding social loss (*t* = −3.22, *p* < 0.01, Cohen’s *d* = −0.51) and potential continuous development in higher age (*t* = 3.01, *p* < 0.01, Cohen’s *d* = 0.49) emerged after wearing the ASS (see left part of Figure 1). For the subscale physical decline, a marginal effect was found in reverse direction (*t* = 2.02, *p* = 0.05, Cohen’s *d* = 0.32).

In an explorative approach, we investigated effects on risk perception, perceived obsolescence and desired support through technology. Participants reported a significantly increased risk perception regarding their susceptibility to age-associated impairments after the ASS intervention (*t* = −2.80, *p* < 0.01, Cohen’s *d* = −0.44) and higher obsolescence (*t* = −2.04, *p* < 0.05, Cohen’s *d* = −0.33; see right part of Figure 1).

With respect to desired support through technology, overall acceptance did not change from baseline to post-intervention. However, participants reported a higher acceptance regarding assistive devices/robots in the household domain after wearing the ASS (*t* = −2.62, *p* < 0.05, Cohen’s *d* = −0.42).

Order effects with respect to the two conditions (geriatric assessments with or without ASS first) were not observed. We additionally examined in bivariate correlation analyses if change scores of those constructs exhibiting significant pre-to-post differences were related to socio-demographic and health related background variables. Overall, change scores in aging-related cognitions (AgeCog subscales continuous growth, physical decline, and social loss), risk perception, and obsolescence were not related to participants’ chronological age, subjective age, gender, BMI, and subjective health with two exceptions: A younger chronological age was related to a more positive shift in the AgeCog physical decline subscale whereas a higher BMI was associated with a larger change towards a higher risk-perception.

Furthermore, we correlated the results of the functional measures with the ASS with the technology acceptance assessment. Participants’ performance in the Short Community Balance and Mobility Scale (Gordt et al., 2020) and the 30-s chair-stand test (Jones et al., 1999) were not related to overall technology acceptance and the three subscales. However, participants who performed worse with respect to the grip strength measure, the Timed up and Go test (Podsiadlo and Richardson, 1991), and the walking-related part of the Short Physical Performance Battery (Guralnik et al., 1994) while wearing the suit, reported (marginally) higher technology acceptance afterwards. Table 3 depicts correlations of physical performance tests with acceptance regarding assistive technology while controlling for age.

**Table 3:** Partial correlations of physical performance tests with desired support through assistive technology.

Variables	TUG^a^	SPPB^b^	Grip^c^	sCBM^d^	30sec Chair^e^
Medication^f^	0.14	−0.36^*^	−0.16	0.27	0.14
Household^f^	0.25	−0.17	−0.29^+^	−0.08	0.10
Body care^f^	0.31^*^	−0.21	−0.29^+^	−0.12	−0.04
Overall technology acceptance^f^	0.29^+^	−0.30^+^	−0.31^*^	0.03	0.09

*N = 40; partial correlations controlling for chronological age*.

a *Timed Up and Go test in seconds, higher scores indicate worse performance/longer time needed;*

b *Short Physical Performance Battery, Walking Score; higher scores indicate better performance;*

c *Grip Strength, higher scores indicate better performance;*

d *Short Community Balance Scale, higher scores indicate better performance;*

e *30-second chair-stand test, higher scores indicate a higher number of rises from the chair;*

f *Desired support through technology; 1–4, higher scores indicate higher agreement*.

+ *p* *< 0.10;*

* *p < 0.05*.

## Discussion

Age simulation interventions are a relatively new but rapidly growing approach (Eost-Telling et al., 2020; Bowden et al., 2021) that mostly aim at enhancing empathy and attitudes toward older adults among young adult sample, i.e., students in health-related professions. As views on aging are very important for diverse developmental outcomes but have not been studied yet in this context, we aimed to address possible effects of ASS interventions on individual and general views on aging in a within-subjects design among middle-aged to young-old adults. As expected, we found that with respect to aging-related cognitions, higher negative expectations regarding social integration and continuous development in higher age emerged after wearing the ASS. Ratings in the physical domain of the AgeCog scales even marginally moved to a more positive perception, which might be due to the expectations participants had when being informed on the ASS and entering the experiment. After the simulation, several participants expressed being relieved that they were still able to master the tasks within the geriatric assessments rather well.

In contrast, awareness of age-related gains and losses (AARC) and general age stereotypes in the domains of leisure/social commitment and physical/health as well as did not change from pre- to post-assessment. As AARC also captures self-referential or individual views on aging like the AgeCog scales, the null finding here might be due to the framing of the concepts, which is also mirrored in the respective wording of item prompts. In the AARC assessment, participants have to refer to real experiences that they have already made (“With my increasing age, I realize that… e. g., my mental capacity is declining.”). An age simulation might not have the power to change those long-term awareness-related processes, whereas for aging-related cognitions (AgeCog: “Ageing means to me that…”) the ASS intervention was able to change those views regarding one’s own aging-process at least in a short time-frame. The null effect on age stereotypes might indicate that ASS rather induce an individual aging experience that is not necessarily generalized to perceptions regarding older people as a social group. According to Levy’s (2009) stereotype embodiment theory, negative general views on aging can be detrimental via self-stereotyping and internalization. The reverse direction, that experimentally induced changes in individual perceptions on aging simultaneously affect general age stereotypes (externalization hypothesis; see Rothermund and Brandtstädter, 2003) does not find support in our data. Overall, our findings with stronger effects in self-referential and short-term views on aging measures point to the importance of situational context. In this vein, Hughes and Touron (2021) call for a more contextualized perspective in research on views on aging, as daily life offers a variety of situational contexts and experiences that directly influence our aging-experiences.

Regarding our broader target constructs, results indicated increased perceptions of obsolescence after the ASS intervention. This points toward the importance of debriefings with opportunities for participants to discuss their experience in order to address negative feelings related to an expected loss of social integration with higher age (Brandtstädter and Wentura, 1994; Kaspar, 2004). Moreover, our data indicated an increase in risk perception or susceptibility to age-associated impairments after the ASS intervention. This shift among our participants from a quite low towards a more realistic (i.e., medium) risk perception might be used as a teachable moment for behavior change (Flocke et al., 2014) in terms of engaging in a healthy lifestyle. Notably, those participants with a higher BMI exhibited an even larger shift in this favorable direction, which might be also valuable for interventions, if results are replicated in larger studies. The desired support through robots and intelligent assistive technology as a measure of technology acceptance was not affected by the ASS intervention as an overall indicator, but an increased acceptance regarding assistive devices/robots in the household domain was observed. When relating performance in the established geriatric assessments while wearing the ASS to technology acceptance, several associations revealed that those with higher difficulties reported higher openness to be supported by intelligent assistive devices.

### Limitations and Strengths

Our study has some limitations that need to be acknowledged. First, due to our relatively small sample size, we were only able to detect at least medium-sized effects, whereas for smaller effects and multivariate analyses, our study was underpowered. As this was the first attempt in the field of age-simulation to study general and individual views on aging, the presented preliminary findings require replication in larger samples. A second limitation is due to the design of the ASS, which strongly focusses on *physical* changes related to the aging process. However, lifespan development unfolds in different domains of functioning and consists of both gains and losses. This multidimensionality and multidirectionality (Baltes et al., 2006) has been depicted in our study with regard to the chosen views on aging assessments, but as the age suit does not intend to induce positive changes and does not mimic for example cognitive impairments or changes in the social–emotional domain, the simulation had an unidimensional approach. Third, our study only focused on pre-to-post effects in a within subjects design and lacked a control group of participants not experiencing an age simulation.

Apart from that, the explicit inclusion of more diverse middle-aged to even young-old adults instead of a young student population and the design combining established questionnaires with validated geriatric assessments with and without the suit are major strengths of our study. We further intended to overcome the limitation of the usually very short time frames for the simulation experiences (i.e., 10 min with the suit; Lee and Teh, 2020). By assessing background characteristics such as health status and BMI and being able to draw on the performance data with and without the suit, we were able to provide a more comprehensive picture.

### Implications and Outlook

For future research, studies with follow-up measurements are needed to address the duration of the found effects. Furthermore, similar within-subject designs would profit from more diverse pre-post-measurements of technology acceptance, technology proficiency, technology-related self-efficacy beliefs, or even technology performance measures (Roque and Boot, 2018; Schmidt and Wahl, 2019), in order to explore more differentiated effects that might be used to facilitate technology adoption in older age. In our study, some positive effects on technology acceptance were found for those participants, who had higher difficulties with the physical assessments with the suit. If these results are replicated and extended to a larger variety of (assistive) technologies and systems, this might be a starting point for interventions designed to facilitate technology adoption.

As this study assessed changeability of views on aging and related constructs via implementation of an ASS for the first time, we chose a highly controlled lab setting and standardized geriatric tests and hence focused on internal validity. A major problem in the previous literature has been that how long and under which conditions (i.e., specific tasks, instructions) the ASS were worn shows large heterogeneity or is not reported in any detail at all. Therefore, we decided to keep the duration of wearing the ASS as well as the series of tasks to be conducted under ASS conditions constant. With respect to ecological validity (i.e., the validity of findings in everyday life settings), assessing task performance in participants’ natural environment, e.g., by including complex activities of daily living such as technology use, might be a next step for future studies. Moreover, the validity of ASS in terms of a realistic simulation of “higher age “should be explored, especially with earlier studies focusing solely on younger participants. In our study, the “manipulation check” of asking participants how old they felt in the ASS turned out to match a simulation of fourth age (80+) on average, but this has to be questioned among younger adults. Hence, carefully designed and controlled validation studies quantifying effects of ASS on physical performance outcomes are warranted.

To sum up, earlier studies concentrated on the potential of age simulation interventions regarding empathy and attitudes towards older adults in student samples, mainly for educational purposes. However, our results in a sample of middle-aged to young-old adults point out that there may be negative effects regarding views on aging, especially in terms of cognitions regarding participants’ own aging process in the domain of social losses and expectations regarding ongoing development and continuous growth while aging. Remarkably, we observed some transfer effects, as significant changes emerged in the non-physical domain, which is the main focus of ASS interventions.

With regard to practical implementations and potential interventions, ASS might have positive effects on technology acceptance in terms of openness towards assistive devices that support everyday activities as well as on motivation to prevent age-associated impairments. We conclude that ASS should be applied in combination with expert supervision and education on losses and gains during the aging process to prevent negative age stereotypes and provide a comprehensive and differentiated picture. Future research should follow rigorous research designs, and consider a larger diversity of participants outside student populations, i.e., in terms of age range, fitness level, experience with and knowledge about technology.

## Data Availability Statement

The raw data supporting the conclusions of this article will be made available by the authors, without undue reservation.

## Ethics Statement

The studies involving human participants were reviewed and approved by the Ethics commission of the Faculty of Behavioral and Cultural Studies at Heidelberg University, Germany. The patients/participants provided their written informed consent to participate in this study.

## Author Contributions

LS initiated and designed the study, raised funding, conducted data analysis and wrote the manuscript. AS assisted in designing the study and reviewed the manuscript. TG collected the questionnaire data, conducted the age simulation intervention, and reviewed the manuscript. H-WW initiated the study, raised funding, and reviewed the manuscript. All authors approved the final version of the manuscript.

## Funding

This study was funded by the Carl Zeiss Foundation (*HeiAge,* Grant No. 0563–2.8/738/2; subproject *Awareness of Aging and Age Simulation*, Heidelberg University, Germany).

## Conflict of Interest

The authors declare that the research was conducted in the absence of any commercial or financial relationships that could be construed as a potential conflict of interest.

## Publisher’s Note

All claims expressed in this article are solely those of the authors and do not necessarily represent those of their affiliated organizations, or those of the publisher, the editors and the reviewers. Any product that may be evaluated in this article, or claim that may be made by its manufacturer, is not guaranteed or endorsed by the publisher.

## Footnotes

^1^ https://osf.io/f9hrp/
